# Relationality: The Role of Connectedness in the Social Ecology of Resilience

**DOI:** 10.3390/ijerph20053865

**Published:** 2023-02-22

**Authors:** Wing Shan Kan, Raul P. Lejano

**Affiliations:** 1 Department of Social Work, Hong Kong Baptist University, Hong Kong 999077, China; 2 School of Culture, Education, and Human Development, New York University, 239 Greene Street, New York, NY 10003, USA

**Keywords:** resilience, social capital, relationality, sustainability, collective action

## Abstract

Previous work has focused on the role of social capital on resilience. However, this research tends to search for civic and other organizations, often formal institutionalized groups which, when they are not found, leads to questions about how social networks are possibly governed. Without formal organizational structures to govern these networks, how is pro-environmental/pro-social behavior sustained. In this article, we focus on a diffused mechanism for collective action, which is referred to as relationality. Relationality is a theory that underscores how social connectedness, through mechanisms of empathy, foster collective action in noncentralized modes of network governance. The concept of relationality addresses important issues not considered by the literature on social capital --so being, we will refer to relational elements as relational capital. Relational capital constitutes a type of asset that communities can activate vis-a-vis environmental and other perturbation. As we describe, the evidence for relationality as an important mechanism for sustainability and resilience is accumulating.

## 1. Introduction

We take up the role of social connectedness in a community’s resilience to environmental shocks (such as extreme weather, climate change, crop infestation). That is, we begin with the observation that the groups of people in a community most impacted by such an even are also often the most socially isolated. And, conversely, we note anecdotal accounts of how connectedness among the people in a community lends them resources to survive, even thrive, even in the middle of such events. The phenomenon of ’social ties’ is one that social network analysis has attempted to elucidate. Often, however, the unidimensional ways by which this literature describes social ties (e.g., as a binary variable) falls short of explaining how social connectedness fosters resilience. 

In this article, we introduce a theory that captures the role of connectedness (or disconnectedness) in resilience (or vulnerability). We refer to this phenomenon as relationality, which has various (related) definitions. Lejano [1] defines relationality as the condition by which individuals (or groups) think and act in coherence with the web of relationships of which they are part. In cognitive terms, connectedness with the other often means that one’s decision-making is no longer directed at one’s individual utility, but also the good of the other. As discussed in detail in Lejano [1], one mechanism by which this occurs is through the emergence of empathy when individuals find a connection. 

One can take stock of the innumerable real-world examples of relationality at work. For example, charities know that making the recipient of charitable donations more tangible and identifiable increases people’s charity, for example [2]. This type of other-regard is found in recent examples of ‘fair trade’ consumerism where organizations strive to make direct linkages between consumer and producer [1]. So we find that building direct interpersonal linkages between ourself and the other increases our capacity for collective action. 

Related, smaller definitions of relationality are also pertinent. Lejano and Kan [3], exploring the role of relationality in the design and enactment of public policy, define relationality in policy life as the manner by which policies are shaped in coherence with the web or relationships among policy actors. As organizational theorists have posited that people make decisions not just out of individual utililty maximization but also based on a logic of appropriateness, Lejano and Kan posit that this logic is none other than consistency with the pattern of inter-individual and inter-group relationships. 

Relationships, of course, can be helpful or harmful, and everything is contextual (which, in part, lies behind the critique of the social capital literature discussed later in this article). But relationships that lead to collective action can exhibit the properties of an ethic of care [4]. Gilligan’s work on ethics in real life suggested that people’s moral decisions are contextual and geared to nurture relationships with the other. Noddings [5]. further describes the characteristics of an ethic of care, namely:

i. interconnectedness, which means no one is excluded from the web of sustaining relationships,
ii. responsiveness, which means taking actions that respond to the needs of the other, and moreover, according to the greatest attention to those with the greatest needs,
iii. agency, which means regarding the other not as a passive object of beneficence but an active agent,

In the context of extreme events and global environmental change, relationality requires connectedness to the most vulnerable and ’disproportionately’ responding to their situation. As we will discuss in the next section, there is much literature on social capital and resilience that captures much of these ideas. Later on, however, we discuss how relationality is a more encompassing and fine-grained concept that addresses the limitations of the social capital literature.

## 2. Social Capital and Resilience

“Individuals and communities resilient to climate change anticipate risks; reduce vulnerability to those risks; prepare for and respond quickly and effectively to threats; and recover faster, with increased capacity to prepare for and respond to next threat” [6] (p. 4592). Social capital plays a significant role in increasing vulnerable communities’ resilience to extreme weather and other disasters including both preparedness and post-recovery [7,8,9]. Community resilience is an important strategy for disaster management [7]. Community resilience refers to a dynamic process of maintaining successful adaption to social, environmental and political stresses, changes and disturbances from communities or group [10]. There are many examples in which social capital has been shown to be a key factor to enhancing communities’ resilience to disasters, such as in Nepal [11], India [12], Vietnam [13], Japan [14], China [15], the UK [16] and the United States [8].

According to Bourdieu [17], social capital is defined as “the aggregate of the actual or potential resources which are linked to a possession of a durable network of more or less institutionalized relationships of mutual acquaintance or recognition” (p. 248). Therefore, social capital is created through social connection and social networks. For Putnam [18], social capital is “features of social organizations, such as networks, norms and trust that facilitate action and cooperation for mutual benefit” (p. 35). Social capital can be a facilitator of interpersonal cooperation which enhances collective norms and trust for promoting collective well-being. 

There are three main types of social capital, which are bonding, bridging, and linking. Each of these types helps vulnerable people evacuate before and after natural disasters in a different way [7,9]. Bonding capital refers to the connections and interaction among people with strong bonds, such as family members and relatives [19]. Strong bonding capital allows vulnerable people to receive emotional support, social support, and personal assistance in disasters, such as warnings, locate shelters and obtain immediate aid, which is important for social cohesion and cooperation. Bridging capital refers to the relationships among people with loose, weak, and external ties, such as friends, community neighborhoods, for example, church collect money for people in need after disaster [20]. Strong bridging capital can assist long-term recovery from disaster by collaboration from different communities to broaden the assets-base. Linking capital refers to the relationships with institutions, such as government departments, non-governmental organizations, which have relative power in the society by providing access to services or resources [19,20]. Strong linking capital connects disaster-affected groups with resources and formal support from the government, such as rebuild their home and obtain business loans for long-term investments after disaster [21]. Resilience is increased by the social capital through improved social connections and social networks [8,22].

Bonding, linking, and bridging social capital represent various kinds of social connections and social networks. Through the mechanisms from bonding capital, bridging capital, and linking capital in promoting collective action to provide supports and services before and after disasters [23]. A more general treatment of resilience from a social-ecological framework is found in Stokols et al. [24], where resilience is associated with the coherent interlinkage of multiple forms of capital. 

## 3. Relationality: Beyond Social Capital

As discussed in the previous section, an important literature exists on the role of social capital in community resilience vis-a-vis extreme weather and global environmental change. However, the concept of social capital, itself, does not have sufficient explanatory power to capture essential, relational elements of resilience. Rather, a focus directly on richly describing the role and action of relationships among actors is needed. While not able to explore this concept and apply it more adequately in this short communication, we can nonetheless sketch the power of this new concept by comparing it against extant notions of social capital. 

Social capital is defined in various ways, as explained above. It is used primarily in two ways however: in the sense of aggregate social networks and organizational assets [25] or as a form of personal, bankable resource that is in a sense capitalizable [17]. In both of these interpretations, the role and action of relationships is not readily tangible. The first sense of it is too macroscopic, as there are countless examples of communities where a myriad array of organizations, agencies, and NGOs exist, but routine services break down. The second sense of it is, for a different reason, also too reductionistic, as relationships are collapsed into a unitary notion of capital (almost like a bankable asset). Only infrequently does an analysis of social capital venture into understanding how the social ties function in context, for example [8]. Breaking down social capital into different sub-types (bonding, bridging, and linking) still falls short of describing, with enough richness, the nature of social and organizational ties and how these relationships function to increase resilience. 

Our use of the term, relationality, is meant to put prime analytic focus on the relationships themselves: describing them and observing how they work from case to case. There are numerous advantages to this framework. First, it emphasizes the need to focus on social (and institutional) connectedness especially among vulnerable members of the population and, secondly, to analyze how these relationships function. 

There is another advantage to the use of the concept of relationality, which is that, in its deepest sense, it does not function simply like an asset or a capital good. As ethicists of care propose, “relationship” entails attending to who the other is and what the other needs. Relationality is grounded on seeing and hearing the other and acting out of empathy, an element that is not necessarily part of extant notions of social capital.

The preceding review of the literature represents a ‘meta-analysis’ of sorts where different bodies of literature converge on a common theme, which is the role of social connectedness on resilience. The flip side of the argument also finds support int he literature, which is the role of disconnectedness on vulnerability. In a review of literature on disasters and social capital, Carmen et al. [26] draw conclusions about how different forms of social capital function to increase resilience. One insight emerging from this analysis pertains to situations when social capital does not function to protect communities, and it is when there are elements of social exclusion. “Empirical studies also suggested that social capital is important but insufficient in shaping resilience for those who are marginalised, excluded or in contexts of high social inequality” and, later on, “This includes a particular focus on social norms of exclusion in limiting the role of social capital in resilience for marginalised social groups” [26], pp. 1380, 1383.

Voss [27] echoes this finding and points to the role of social exclusion in reducing the discursive agency of the vulnerable to make their voice heard and exercise agency: “If their interests and their definitions of the world—including own definitions of risk and normality—remain structurally unheard, people’s ability to manage everyday routine, but even more their ability to deal with extreme situations declines” [27], p. 41.

It is on the strength of these overlapping bodies of scholarship that we make the case, in a qualitative way, of the importance of social connectedness to resilience (and, conversely, of disconnectedness to vulnerability).

Table 1 differentiates the concept of social capital, as treated in the resilience literature, from that of relationality. This differentiation emerges from the critical theoretic analysis employed herein. Embedded in extant treatments of social capital is a structural assumption, in that it functions as an element of co-determinant of power. In contrast, relationality is an agentic concept, as it traces how interpersonal ties work in the context of particular relationships. While the first concept objectifies social capital as measures of social aggregates (e.g., civic associations), relationality strives to examine relationships as intersubjective interactions. While the first concept implicitly (often) reductionistically assumes an equivalence with conventional capital, relationality differentiates and examines the working and reworking of relationships across particular ties.

## 4. Illustration

### 4.1. Disconnectedness and Vulnerability: Katrina and Sandy

The literature on vulnerability and disasters points to a number of factors that often render populations most vulnerable: age and infirmity, disadvantaged minority status, and social isolation [28]. Of the demographic trends emerging in this century, the social isolation of the elderly is among the most serious, for example [29]. 

These factors were present during Hurricane Katrina (which struck New Orleans on 29 August 2005), where two-thirds of the casualties were aged 65 or older [30]. Health and physical impacts of Katrina were highly correlated with age (i.e., elderly), gender (i.e., female), and indicators of social isolation/integration such as separation from family, membership in organizations, and community bonds [31]. Other studies implicated the social isolation of vulnerable elderly, for example [32]. This element of social isolation was exacerbated by practices of racial segregation, as there were accounts of Black residents seeking help but being turned away from mostly White districts [33].

These factors were present during Superstorm Sandy (which struck New York City on 29 October 2012), were nearly half the fatalities were aged 65 and older. Post-event, some of the most affected were elderly residents of the Red Hook public housing, many trapped in apartments for days without running water and electricity as the storm surge wiped out electrical systems and pumps (frequently located in building basements). As with the experience with Hurricane Katrina, the social and physical isolation of the Red Hook residents increased their level of distress in the aftermath of the storm. These conditions, namely the intersection of physical vulnerability with social disconnectedness is, in fact, a condition that is perhaps growing in many major world cities [34].

### 4.2. Connectedness and Resilience: Bangladesh Cyclone Preparedness Programme

The cycle of marginalization, isolation, and vulnerability can be turned on its head, however, by building relationships that increase resilience among the vulnerable. This is the case in Bangladesh, which has been disproportionately beleaguered by disaster and the site of the worst natural disaster in history, the Bola Cyclone of 1971, where at least 300,000 people lost their lives [35]. As with the cases above, ample literature attest to the role played by poverty, amplified by social isolation, in fatalities and injuries from cyclones in Bangladesh. For example, some researchers point to the role of social exclusion of lower-income females from channels of social contact and communication in the higher rate of fatalities among women in rural villages, for example [36,37].

Since the tragedy of 1971, however, government agencies and nonprofits in Bangladesh have collaborated in creating a distinctive new agency, the Cyclone Preparedness Programme, that at least anecdotally has been a key reason behind the much-reduced fatalities from cyclones in the ensuing decades, for example [38].

The CPP’s design is based on many principles of relationality. It does not simply conceive of the vulnerable as objects of risk prevention but active agents of change. For this reason, more than 90% of the CPP’s workforce is composed of community volunteers. In addition, the CPP is close to achieving its goal of having the CPP corps be at least 50% female in composition, with the attendant goal of increasing participation and leadership of women in the community in the disaster reduction efforts [38].

The CPP’s training workshop for volunteers is an example of relationality translated into practice [39,40]. Consonant with the idea of community residents being active agents of change, the workshop trains residents in being expert risk communicators in the community. This requires translating technical bulletins into the form of everyday narratives that they can then disseminate in the community. It also allows others in the community, further down the chain of communication, to likewise participate as risk communicators. It also requires relationship-building, translating into volunteers often going door to door to ensure no one is left outside the network for disaster risk prevention. At a meeting of the CPP managers and partners in 2018, participants spoke about bringing risk communication messages to mosques, markets, and other public places. One participant (a representative from UN Women), further impressed the idea of relationality when she spoke up and added: “This is a good idea, to bring knowledge about risks like tropical cyclones to the marketplace, to the mosque, to the schools... But what about many women in Bangladesh, when the women are not to be found in the market, or the mosque, or the schools? How do we reach them when many do not or cannot even go outside their home? If we want a program that will make change, we have to reach these women who are traditionally excluded from these places and these programs” (Dilruba Haider, UN Women’s Program Specialist, as transcribed by one of the authors, who participated in this symposium).

These relational ideas are founded upon seeing the other, especially the most vulnerable other, not in objective terms but as equal partners in resilience. Thus, residents comprise a major part of the disaster preparedness response, and their voice and agency recognized. 

## 5. Conclusions

This article discusses a new concept, “relationality”, which underscores how social connectedness, through mechanisms of empathy, foster collective action in promoting sustainability and resilience. “Relationality" also addresses the limitations of the social capital literature. Even though social capital plays an important role in community resilience vis-a-vis extreme weather and global environmental change, the concept itself does not have sufficient explanatory power to capture relational elements of resilience. In other words, the concept of social capital is unable to explain how the role and action of relationships among actors can increase resilience. 

Our review of different bodies of literature illustrates how “relationality” is an important mechanism for enhancing sustainability and resilience. As a mode of analysis, “relationality” suggests that analysts more richly describe the pattern of relationships among actors through the emergence of empathy, especially the most excluded and vulnerable members of a community. The advantages of this framework include, (1) emphasizing the need to focus on social and institutional connectedness; and (2) analyzing how different social and organizational ties’ function. It is argued that patterns of relationships are highly contextual, in ways not captured by the notion of social capital. As a prescription for institutional reform, relationality suggests building strong, functioning ties with all members of a community, and building mutual processes of collaborative support through them.

## Figures and Tables

**Table 1:** Mapping Concepts: Social Capital vs. Relationality.

Social Capital	Relationality
structure	agency
objectivism: social aggregate	phenomenology: intersubjective relationships
equivalence: fungibility with capital	differentiation: relational contextuality

## Data Availability

Not applicable.
